# Local and Travel-Associated Transmission of Tuberculosis at Central Western Border of Brazil, 2014–2017

**DOI:** 10.3201/eid2703.203839

**Published:** 2021-03

**Authors:** Katharine S. Walter, Mariana Bento Tatara, Kesia Esther da Silva, Flora Martinez Figueira Moreira, Paulo Cesar Pereira dos Santos, Dândrea Driely de Melo Ferrari, Eunice Atsuko Cunha, Jason R. Andrews, Julio Croda

**Affiliations:** Stanford University School of Medicine, Stanford, California, USA (K.S. Walter, K.E. da Silva, J.R. Andrews);; Federal University of Grande Dourados, Dourados, Brazil (M.B. Tatara, F.M.F. Moreira, P.C.P. dos Santos, D.D. de Melo Ferrari);; Central Laboratory of Public Health, Campo Grande, Brazil (E.A. Cunha);; Federal University of Mato Grosso do Sul, Campo Grande, Brazil (J. Croda);; Oswaldo Cruz Foundation, Mato Grosso do Sul, Brazil (J. Croda);; Yale School of Public Health, New Haven, Connecticut, USA (J. Croda)

**Keywords:** tuberculosis and other mycobacteria, transmission, genomic epidemiology, migration, borders, incarceration, Brazil, bacteria

## Abstract

International migrants are at heightened risk for tuberculosis (TB) disease. Intensified incarceration at international borders may compound population-wide TB risk. However, few studies have investigated the contributions of migration, local transmission, or prisons in driving incident TB at international borders. We conducted prospective population-based genomic surveillance in 3 cities along Brazil’s central western border from 2014–2017. Although most isolates (89/132; 67%) fell within genomic transmission clusters, genetically unique isolates disproportionately occurred among participants with recent international travel (17/42; 40.5%), suggesting that both local transmission and migration contribute to incident TB. Isolates from 40 participants with and 76 without an incarceration history clustered together throughout a maximum-likelihood phylogeny, indicating the close interrelatedness of prison and community epidemics. Our findings highlight the need for ongoing surveillance to control continued introductions of TB and reduce the disproportionate burden of TB in prisons at Brazil’s international borders.

The global expansion and local spread of tuberculosis (TB) have been shaped by patterns of human migration (*1*–*4*). The 258 million international migrants who live outside their country of birth are frequently put at high risk for TB disease and death because of the many health risks associated with migration including limited access to healthcare (*5*,*6*). Further, in countries with low- or medium-incidence of TB, a substantial proportion of TB is frequently found among recent immigrants (*7*,*8*). Understanding the contribution of local transmission and importation of *Mycobacterium tuberculosis* acquired elsewhere to incident TB cases can inform public health responses. However, few studies have explored the drivers of incident TB along international borders.

Brazil’s national borders, settings characterized by frequent population movement and often overburdened health systems, have higher TB incidence than do nonborder areas (*9*–*11*). In Mato Grosso do Sul state in the Central West region of Brazil, TB notification rates, mortality rates, and rates of treatment abandonment are higher in counties at the borders with Bolivia and Paraguay, compared with counties in the state’s interior (*11*). Similarly, rates of drug resistance and multidrug resistance are higher at the state’s border than elsewhere in the state (*12*). However, the drivers of the increased incidence of TB here remain unknown. The long and variable latency period of TB makes it difficult to identify where transmission occurred.

To reduce the burden of local transmission, identifying congregate settings that play a disproportionate role in transmission is an urgent priority. TB notification rates have rapidly increased within prisons in Brazil (*13*), and TB is increasingly concentrated among incarcerated populations. In Mato Grosso do Sul, the state with the highest incarceration rate in Brazil (618/100,000 population) (*14*), 28.9% of notified TB cases occurred among incarcerated persons in 2017. Prisons are not isolated institutions, and frequent movement of persons inside and outside prisons means that the heightened TB risk created by prison environments may extend to nearby communities (*15*). Furthermore, prisons are frequently high-transmission environments for drug-resistant TB (*16*,*17*). Although extremely drug-resistant TB (XDR TB) is thus far less prevalent in prisons in Brazil than those in Eastern Europe, pre-XDR TB has been associated with prisons in the southern state of Rio Grande do Sul, Brazil (*18*). Whether prisons similarly amplify drug-resistant TB in border cities is not known.

To investigate the drivers of TB transmission along Brazil’s borders, we conducted a prospective genomic epidemiology study of *M. tuberculosis* in the 3 largest international border cities in Mato Grosso do Sul, Brazil. We assessed the phylogenetic structure and predicted transmission clusters to characterize the contribution of local transmission and migration-associated importation to incident TB cases and the role of prisons in driving local transmission.

## Methods

### Study Population and Data Collection

We conducted a population-based prospective study of newly diagnosed and retreated pulmonary TB cases in 3 cities at Brazil’s borders with Paraguay and Bolivia during January 2014–April 2017. Patients with clinical suspicion of TB sought care at primary care providers or hospitals in Ponta Porã, Corumbá, and Ladário, Brazil. Diagnostic tests were done at Hospital Regional Dr. José de Simone Neto in Ponta Porã and the Laboratório Municipal de Corumbá, a public health diagnostic laboratory. Patients with nontuberculous mycobacteria or without positive cultures for *M. tuberculosis* were excluded. There was no incentive for study participation. After a positive culture, a team of researchers carried out a home or prison visit to recruit participants and administer the study questionnaire. Participants answered structured questions about their birthplace; residential address and residential history; previous history of TB diagnosis, treatment, and treatment outcomes; potential contact with patients who had pulmonary TB; incarceration history (incarcerated at the time of diagnosis, formerly incarcerated, any contact with those incarcerated, or no contact with incarcerated persons); and travel history. Recent immigrants were defined as participants with residency in Brazil for <2 years; recent travel was defined as international travel of any duration in the previous 5 years. We obtained additional sociodemographic and clinical data from the National Reporting System on Notifiable Diseases (SINAN). We stored and managed data in an electronic database (REDCap, https://projectredcap.org). We used a 3-sample proportion test to determine whether study participants were representative of all notified TB cases with respect to incarceration status reported in SINAN; we compared the proportion incarcerated, not incarcerated, and with no information about incarceration status at the time of TB notification among study participants and all notified TB cases. We were not able to do a similar comparison for immigration history or recent travel, which were collected in questionnaires.

All participants provided written consent. We obtained approval from the Research Ethics Committee of the Federal University of Grande Dourados (no. CAE 12676613.3.1001.5160) and Stanford University Institutional Review Board (IRB-40285).

### Laboratory Diagnosis and Drug Susceptibility Testing

All sputum specimens collected in the participating laboratories were examined by microscopy or GeneXpert MTB/RIF (Cepheid, https://www.cepheid.com), processed with sodium hydroxide (NaOH), and inoculated in Ogawa-Kudoh culture medium. We incubated cultures at 37°C for <8 weeks and checked weekly for visible colonies at the participating laboratories. We determined microbial species using the MPT64 protein detection-based immunochromatographic rapid test (SD Bioline Kit; Standard Diagnostics, Inc., (http://www.standardia.com). We performed phenotypic susceptibility testing of *M. tuberculosis* isolates using the BACTEC MGIT 960 system (Becton Dickinson, https://www.bd.com)

### Whole-Genome Sequencing

We extracted DNA from cultured isolates with the manual CTAB (cetyl trimethylammonium bromide) method and sequenced the whole genome on an Illumina NextSeq (2 × 151-bp) (https://illumina.com). Sequence data are available on the Sequence Read Archive (accession no. PRJNA671770). We trimmed low-quality bases (Phred-scaled base quality <20) and removed adapters with Trim Galore (stringency = 3) (*19*). We used CutAdapt to further filter reads (–nextseq-trim = 20 – minimum-length = 20 – pair-filter = any) (*20*). To exclude potential contamination, we used Kraken2 (*21*) to taxonomically classify reads and removed reads that were not assigned to the *Mycobacterium* genus or that were assigned to a *Mycobacterium* species other than *M. tuberculosis*.

We applied variant calling methods closely following those described in Menardo et al. (*22*) to be consistent with the methods used for molecular clock estimation. We mapped reads with bwa version 0.7.15 (*23*) (bwa mem) to the H37Rv reference genome (NCBI accession no. NC_000962.3) and ﻿ performed local read realignment with the RealignerTargetCreator and IndelRealigner modules of GATK version 3.8. We created read pileups with Samtools version 1.9 and called variants for individual samples with varscan version 2.4.4. As described by Menardo et al (*22*), we called variants at positions with a minimum mapping quality of 20; minimum base quality of 20; minimum read depth of 7×; minimum percentage of reads supporting the call 90%; and <90%, or <10% of reads supporting a call in the same orientation (varscan strand bias filter).

We excluded single-nucleotide polymorphisms (SNPs) in previously defined repetitive regions (PPE and PE-PGRS genes, phages, insertion sequences, and repeats longer than 50 bp) (*24*). We excluded all isolates with mean coverage <15× ﻿and isolates with >50% of SNPs failing the strand bias filter, and genomes with >50% of SNPs that had a variant allele frequency of 10%–90%. We also excluded isolates that were assigned to multiple lineages with TBProfiler version 2.8.6 (*25*). We measured drug-resistance associated mutations with MykrobePredict version 0.8.0 (*26*), using the 201901 database of genomic predictors of resistance (*27*). We identified lineage with TBProfiler version 2.8.6 (*25*).

### Phylogenetic and Bayesian Evolutionary Analysis

We constructed full-length consensus sequences from VCF files and used SNP sites to extract a multiple alignment of internal variant sites (*28*). We used the R package ape version 5.4 to measure the number of pairwise site differences between samples (*29*). We fit maximum likelihood trees with RAxML-ng 0.9.0 (*30*). We used a general time-reversible substitution model and a Stamatakis ascertainment bias correction for invariant sites in our alignment. We divided the number of invariant sites by 1,000 to avoid issues created by small branch lengths. We defined nucleotide stationary frequencies as frequencies in the reference genome. We clustered isolates using a common 12-SNP threshold (*31*) for relatedness of isolates from epidemiologically related hosts. We constructed haplotype networks with the R package pegas version 0.13 (*32*).

We fit a Bayesian tree to the sequences from the multidrug resistance (MDR)-associated transmission cluster with BEAST 2.6.2 (*33*). We applied a strict clock and constant coalescent population size model and used TB notification dates to calibrate tips. We used an HKY substitution rate model and estimated base frequencies. Because it would not be possible to estimate substitution rates from a small tree, we specified a narrow log-normal prior distribution on substitution rate (mean −16.1, SD 0.16), consistent with previous estimates of *M. tuberculosis* lineage 4 substitution rate estimates (mean 5.8 × 10^−8^, SD 2.0 × 10^−8^) (*22*). We ran sampling chains for 100 million iterations or until effective sample size estimates were >200 (Tracer version 1.7.1 [*34*]), indicating good convergence, and discarded 10% of samples as burn-in. We corrected for ascertainment bias by specifying the number of invariant sites in the alignment.

## Results

### Study Population

**Figure 1:**
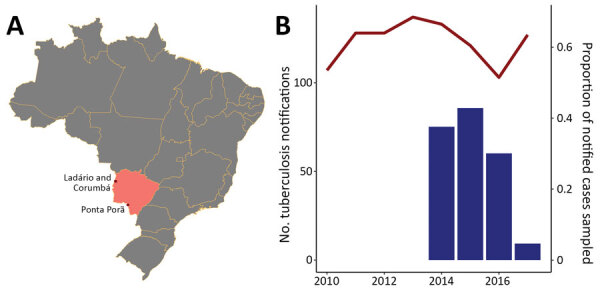
Tuberculosis case notifications in border cities in Mato Grosso do Sul state, Brazil. A) Location of Mato Grosso do Sul state (pink); dots indicate the 3 largest border cities, Ponta Porã, Corumbá, and Ladário, which is surrounded by Corumbá. B) Tuberculosis notifications in the 3 border cities reported in the state tuberculosis registry, SINAN, from 2010–2018 (red line), and the proportion of yearly notified cases sampled (blue bars) in this study, January 2014–April 2017.

A total of 400 patients had notified TB in 3 cities at Brazil’s central western border with Paraguay and Bolivia, Ponta Porã, Corumbá, and Ladário, during January 2014–April 2017 (Figure 1). Of these, 243 were cultured and 215 were culture positive. We enrolled 142 participants, and we generated high-quality sequences for 132 (61.4% of culture-positive notifications). Twenty-seven percent of participants reported international travel within the past 5 years (38/142). Fifty-one percent (74/142) of the study population did not have an incarceration history; 18.8% (27/142) were formerly incarcerated; 9.0% were incarcerated at the time of notification (13/142); 7.6% (11/142) reported contact with incarcerated population; and 13.2% (19/142) did not provide information about incarceration history. The proportion of study participants with an incarceration history did not differ significantly from that of the population with notified TB during the study period (p = 0.1585, as determined by 3-sample test for equality of proportions) (Table).

**Table:** Characteristics of study participants with notified tuberculosis, Central West region, Brazil, 2014–2017*

Characteristic	Participants, N = 142
City	
Corumbá	114/142 (80.3)
Ladário	3/142 (2.1)
Ponta Porã	25/142 (17.6)
Sex	
M	92/142 (64.8)
F	50/142 (35.2)
Age, y	
Median (IQR)	37 (27–53)
0–14	1/142 (0.7)
15–20	7/142 (4.9)
21–40	74/142 (52.1)
41–60	44/142 (31.0)
>60	16/142 (11.3)
Race, self-reported	
White	30/141 (21.3)
Mixed	66/141 (46.8)
Black	38/141 (26.9)
Indigenous	7/141 (5.0)
Asian	0/141 (0.0)
Education level, y	
<1	11/133 (8.3)
1–4	47/133 (35.3)
5–8	44/133 (33.1)
9–11	29/133 (21.8)
>11	2/133 (1.5)
Marital status, married	61/134 (45.5)
Monthly individual income <$100 US	104/119 (87.4)
Recipient of a cash transfer program	46/142 (32.4)
International travel within the past 5 y	38/142 (26.8)
Vulnerable population	
Currently incarcerated	16/142 (11.3)
Formerly incarcerated	27/142 (19.0)
Contact with someone with incarceration history	13/142 (9.1)
Homeless	3/142 (2.1)
Immigrant: <2 y resident in Brazil	5/133 (3.7)
Comorbidities	
Alcoholism	33/134 (24.6)
Diabetes	7/134 (5.2)
HIV	8/138 (5.8)
Mental illness	2/134 (1.5)
Hypertension	36/131 (27.5)
Renal failure	31/133 (23.3)
Hepatic failure	34/132 (25.7)
Smoking history	80/140 (57.1)
Drug use	35/134 (26.1)
TB history	
Previous TB	29/141 (20.6)
History of contact with TB case	69/132 (52.3)
BCG scar	104/133 (78.2)
Supervised treatment	41/135 (30.4)

*Values are no. (%) except as indicated. BCG, Bacillus Calmett-Guérin.

All of the 132 whole genome sequences were assigned to the European-American lineage and specifically to sublineages 4.1 (47), 4.3 (71), 4.4 (9), 4.8 (3), and 4.9 (2) with TBProfiler (*25*). Genomic (*19*) and phenotypic predictions of drug resistance were largely concordant: >93% concordance for ethambutol, isoniazid, and rifampin and 75% for streptomycin. For clarity, we refer to the genomic resistance predictions, for which we had information about additional drugs. Whereas most samples (80.3%; 106/132) were susceptible to all drugs, the remaining samples were resistant to >1 drug. A total of 22 (16.7%) isolates were isoniazid resistant, and 3 isolates (2.2%) were multidrug resistant, resistant to both isoniazid and rifampin. Five of the 14 isoniazid monoresistant case-patients and 3 of the 5 isolates resistant to both isoniazid and streptomycin had been previously treated; the 3 MDR isolates were from newly notified cases.

### Phylogenetic Structure

**Figure 2:**
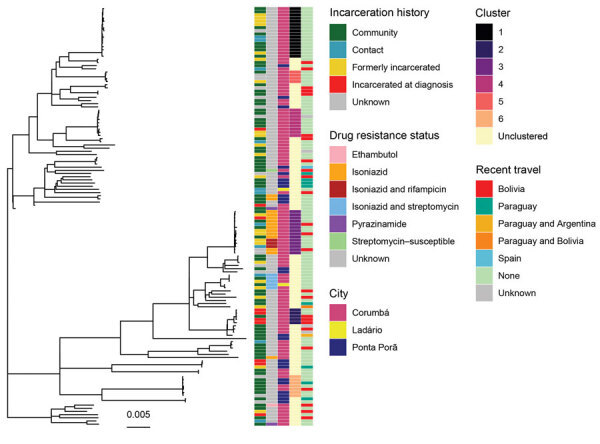
Unrooted maximum-likelihood phylogeny of 132 *Mycobacterium tuberculosis* isolates from Central West Brazil, 2014–2017, inferred from a multiple alignment of 6,590 single-nucleotide polymorphisms. From left, columns are colored by patient’s incarceration history, drug-resistance status, city, predicted transmission cluster, and recent travel history. Incarceration history is defined by responses to the study questionnaire and incarceration information in the tuberculosis registry; community includes patients who have not been incarcerated at the time of tuberculosis notification; contact indicates any reported contact with incarcerated persons; formerly incarcerated includes patients who report incarceration prior to their tuberculosis notification; and incarcerated at diagnosis includes patients notified at time of incarceration. Transmission cluster membership is shown for clusters with >4 isolates; all other isolates are labeled as unclustered. Scale bar indicates substitutions per site.

A maximum-likelihood tree constructed from a multiple alignment of 6,590 SNPs shows a pattern of extensive co-circulating *M. tuberculosis* diversity with several genetically distinct clades of closely related isolates (Figure 2). Isolates from patients with an incarceration history (incarcerated at the time of diagnosis or formerly incarcerated) are dispersed throughout the tree and do not form a monophyletic clade. Neither do isolates from community members reporting no incarceration history, indicating a lack of distinct epidemics within and outside prisons. Four isolates were from recent immigrants to Mato Grosso do Sul state; these isolates were similarly distributed throughout the tree and differed by 165–274 SNPs.

We observed evidence of limited geographic structure. Isolates from Ponta Porã often form monophyletic clades dispersed throughout isolates sampled from the other 2 cities, indicating that both local transmission and between-city migration contribute to the spread of *M. tuberculosis* strains (Figure 2).

### Transmission Clusters

**Figure 3:**
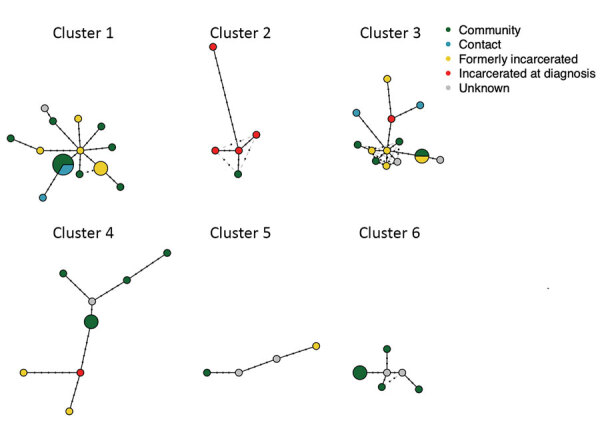
Haplotype networks of the 6 predicted tuberculosis transmission clusters with >4 members from Central West Brazil, 2014–2017. Nodes represent unique haplotypes and are scaled to size. Points along branches indicate single-nucleotide polymorphism distances between isolates. Node color indicates incarceration status at the time of diagnosis. Light gray lines indicate possible alternative links between haplotypes.

To investigate potential recent transmission, we applied a commonly used 12-SNP threshold (*31*) to genetically cluster isolates. We identified 20 clusters, including 89 isolates, and 43 unique isolates. We predicted that if prison and community-associated epidemics were distinct, isolates from the community would be most closely related to and cluster with other isolates from the community. Conversely, if transmission frequently occurred between incarcerated and nonincarcerated persons, we would expect no clear genetic differentiation between isolates from the community and prisons. Of the 20 clusters, 9 included participants both with and without a reported incarceration history, 10 included only participants with no reported incarceration history, and 1 included only persons who were currently or formerly incarcerated (Figure 3).

To test for assortative clustering between participants with an incarceration history and without an incarceration history, under which participants disproportionately cluster with others with the same incarceration status, we applied a permutation test. We randomly reassigned reported incarceration histories to the observed clusters 1,000 times, holding the number and size of clusters constant. The observed number of clusters containing members with and without an incarceration history does not significantly differ from clustering under proportionate, or random, mixing (p = 0.19). Similarly, the observed number of clusters including only members currently or formerly incarcerated does not differ from what would be expected under proportionate mixing (p = 0.76). The observed patterns of clustering indicate that transmission networks inside and outside prisons are closely related.

To further investigate genetic structure among sampled isolates, we identified the most closely related isolate to each study isolate, including multiple isolates when there were multiple nearest genetic neighbors. Many isolates from participants reporting no incarceration history were most closely related to isolates from participants who were currently or previously incarcerated (15/62; 24.2%) or participants reporting contact with those incarcerated (7/62; 11.3%) (Figure 3). Similarly, isolates from participants with an incarceration history were most closely related to isolates from participants reporting no incarceration history (18/38; 47.4%). The close relatedness between many isolates from participants within and outside prisons again suggests potential transmission between those with an incarceration history and those outside of prison. TB transmission rates are elevated in prisons compared with rates outside of prisons (*13*,*35*); although we cannot infer the direction of transmission from genomic clusters alone, the close interrelatedness of prison and community epidemics indicates that prison epidemics can affect the community.

Patients with a recent history of travel, defined as travel within the previous 5 years, were significantly more likely to be infected with an unclustered or unique isolate (17/36) than patients with no history of travel (25/92) (p = 0.03 by Fisher exact test; odds ratio = 2.38), potential evidence that they were infected outside of Brazil. These participants reported travel to Bolivia (9), Spain (1), Paraguay (5), Paraguay and Argentina (1) and Paraguay and Bolivia (1). All 4 isolates from recent immigrants to Brazil were unique and fell outside of predicted transmission clusters; again, possibly indicating they were infected outside of Brazil. However, limited sampling constrains our ability to predict whether genetically unique isolates represent imported lineages or locally circulating but unsampled lineages.

We then examined the distribution of drug resistance across predicted transmission clusters. The 22 isoniazid-resistant isolates occurred within 4 distinct transmission clusters; 1 isolate fell outside of the clusters, suggesting that isoniazid resistance has emerged or been introduced several times within the sampled isolates. The genetic clustering of resistance indicates that the majority of isoniazid resistance in this study was transmitted rather than acquired de novo*.* Similarly, streptomycin-resistant isolates occurred in 2 predicted transmission clusters, each including 1 person incarcerated at the time of TB diagnosis and a single unique isolate, which was evidence of transmitted resistance. In contrast, the 2 pyrazinamide-resistant isolates were unique.

### MDR-Containing Cluster

The 3 MDR isolates fell within a single predicted transmission cluster of 14 isolates from a single city, Corumbá (Figure 3, cluster 3). All isolates within the cluster were isoniazid resistant and shared the *inhA* S94A mutation. In addition, the 3 MDR isolates all shared the *rpoB* S450L, rifampin-conferring mutation. Two of the MDR isolates occurred among persons who were previously incarcerated, and one was from a participant with an incarcerated family member. The mean pairwise distance between MDR isolates was 4 SNPs (range 3–5 SNPs), suggesting that MDR was transmitted (primary MDR) rather than acquired de novo.

**Figure 4:**
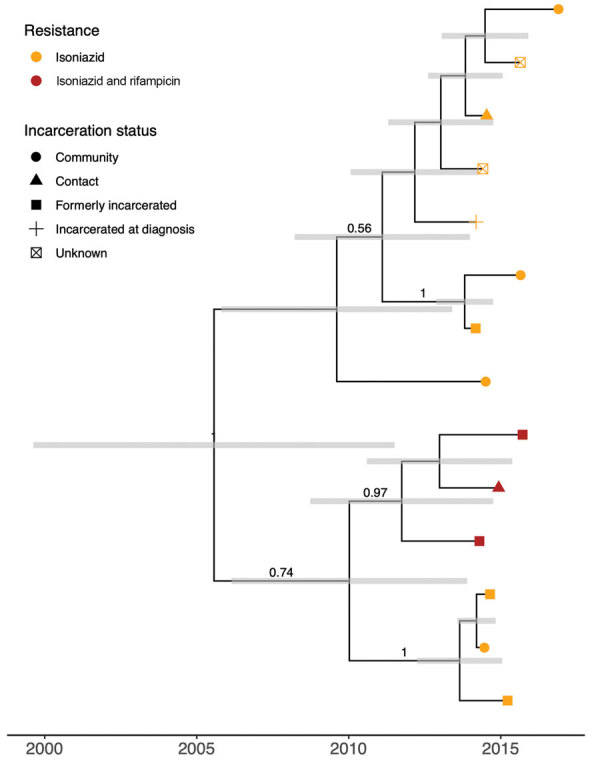
Bayesian timed tree of 14 isoniazid-resistant isolates that circulated for 11 years, from 2005 (95% CI 1998–2010) to November 2016, the most recent sampling date, in Central West region, Brazil. Tip points are colored by genotypic drug resistance; point shape indicates incarceration status. Gray error bars indicate 95% Bayesian highest posterior density intervals for node date. Clade posterior support values are shown on the middle of branches for clades with posterior support >0.5.

We more closely examined the MDR-containing cluster by fitting a Bayesian timed tree (Figure 4). The most recent common ancestor (MRCA) of the cluster occurred in 2005 (95% CI 1998–2010). The MDR isolates fall within a well-supported monophyletic clade, with MRCA in 2011 (95% CI 2008–2014), evidence that MDR evolved a single time among sampled isolates and that MDR TB has been circulating locally for >6 years (the time between the MRCA of the MDR clade and the most recent date of sampling).

## Discussion

In recent years, while TB incidence declined nationally in Brazil, TB notifications have increased in its Central West region border cities. We found evidence that both local transmission and travel-associated introductions contribute to incident cases. In addition, we found that many genomic clusters involved persons with and without an incarceration history, evidence that prison and community epidemics of TB are closely interrelated in cities at Brazil’s Central West border. During 2005–2017, the state’s incarcerated population more than doubled (increasing from 8,273 to 16,634) (*36*). Dramatic rises in incarceration rates, combined with the elevated TB incidence rate within prisons, are likely contributing to ongoing local transmission at the border.

The prevalence of primary isoniazid resistance and MDR TB have increased across Brazil over the past 20 years (*37*). In this border setting, most drug-resistant isolates fell within predicted transmission clusters, indicating that interventions are needed to prevent the ongoing local transmission of drug-resistant strains. Whereas the prevalence of drug resistance in Central West Brazil has not yet reached the levels found in Rio de Janeiro, for example, we found evidence of ongoing local transmission of an isoniazid-resistant clone for >10 years. We additionally identified the emergence of an MDR *M. tuberculosis* clone associated with prisons that circulated locally for >6 years. Our findings highlight the critical need for the early detection of drug-resistant TB to prevent ongoing transmission.

Our investigation of TB transmission at Brazil’s Central Western international borders has several limitations. In a setting characterized by frequent population movement, it is possible that many persons are not linked to healthcare, and TB may be undiagnosed, unnotified, or notified elsewhere. Further, more complete sampling among notified cases would enable a more complete portrait of transmission in border cities. For example, additional sampling could reveal that isolates we identified as genetically distinct do indeed fall within local transmission clusters; our estimates of the contribution of ongoing local transmission are likely conservative. In addition, selection bias could have been introduced if enrolled culture-positive participants were demographically different from the total population with TB. Although we did not find a difference in the proportion of incarcerated patients among study participants and all notified TB patients during the study period, we were unable to compare other characteristics such as recent travel history or migration history. It is possible that recent immigrants may have limited access to healthcare and therefore were undersampled; if so, the result would be underestimation of the role of travel-associated importation in incident TB. More complete information about study participants’ residential and travel histories could inform inferences of where transmission occurred. By contextualizing the *M. tuberculosis* diversity observed within this study with a larger sample of genomes sampled from across Mato Grosso do Sul state, Paraguay, and Bolivia, we could better characterize the contributions of local transmission and importation of lineages into Brazil’s border cities. Finally, because of incomplete epidemic sampling and within-host diversity, phylogenetic trees constructed from consensus genomes do not represent actual transmission histories, but instead, the evolutionary histories of sampled *M. tuberculosis*. Phylogenetic trees enable us to characterize genetic structure in our study sample yet cannot be used to directly assess the probability of individual transmission events nor to quantify the role of high-transmission environments in driving the local epidemic.

Our findings indicate that both local transmission and long-distance importation of TB drive local TB incidence in Brazil’s Central Western border cities. Prison and community TB epidemics are interrelated, and prisons are associated with ongoing transmission of drug-resistant strains. The conditions for transmission and spread of TB in these border communities and congregate settings may undermine the broader national progress in TB control. Our study highlights the need for heightened surveillance and transmission-blocking interventions to prevent continued transmission of drug-sensitive and drug-resistant TB strains.
